# Toxicity, Behavioral Effects, and Chitin Structural Chemistry of *Reticulitermes flaviceps* Exposed to *Cymbopogon citratus* EO and Its Major Constituent Citral

**DOI:** 10.3390/insects13090812

**Published:** 2022-09-06

**Authors:** Chunzhe Jin, Hui Han, Yongjian Xie, Baoling Li, Zhilin Zhang, Dayu Zhang

**Affiliations:** 1 College of Advanced Agricultural Sciences, Zhejiang A&F University, Hangzhou 311300, China; 2 Hubei Key Laboratory of Quality Control of Characteristic Fruits and Vegetables, Hubei Engineering University, Xiaogan 432000, China

**Keywords:** *Reticulitermes flaviceps*, *Cymbopogon citratus*, essential oil, citral, walking and gripping behavior, chitin structure

## Abstract

**Simple Summary:**

*Reticulitermes flaviceps*, as a main wood-boring pest, causes economically significant damage to wood materials. In this study, gas chromatography–mass spectrometry was used to detect and characterize the chemical constituents of lemongrass essential oil (*Cymbopogon citratus* (DC.) Stapf.), and we evaluated the associated vapor insecticidal effect on *R. flaviceps* worker adults. Lemongrass EO and its major constituent, citral, presented significant vapor toxicity against *R. flaviceps*, where the walking and gripping abilities of treated insects were reduced. Chitin content, thermal stability, and crystallinity were also reduced in the treated worker adults. The results of this study suggest that lemongrass EO can potentially be used to develop eco-friendly natural remedies for the management of *R. flaviceps*.

**Abstract:**

Botanical pesticides are considered the most promising alternative to synthetic pesticides, considering their less negative impacts on the environment and human health. Here, we analyzed the components of lemongrass *Cymbopogon citratus* essential oil (EO) and evaluated its vapor activity against *Reticulitermes flaviceps*, in terms of the walking and gripping abilities of workers. In addition, the effects of lemongrass EO and its major component on the cuticular content and structure of chitin in termites were also observed. Our results indicate that cis-citral (36.51%) was the main constituent of lemongrass. In the vapor toxicity assay, the LC_50_ values of lemongrass EO and citral were 0.328 and 0.177 μL/L, respectively. When worker antennae were treated with lemongrass EO and citral, their walking and gripping capabilities were significantly inhibited. In addition, the cuticular content, thermal stability, and crystallinity of chitin in the termites were decreased after treatment with citral. Collectively, this study provides a basis for developing and utilizing lemongrass and citral as a new environment-friendly insecticide resource to control *R. flaviceps*.

## 1. Introduction

Termites are important agricultural and forestry pests in tropical and sub-tropical regions, which damage crops, forests, garden trees, houses, and ancient buildings, thus causing an economic loss of more than USD 40 billion annually worldwide [1]. *Reticulitermes flaviceps* is widely distributed in China, and causes losses for the Chinese economy [2]. Synthetic pesticides are currently the most commonly used method for termite control, which often have a negative impact on the natural environment [3]. Due to the low toxicity, biodegradability, and environmentally friendly nature of essential oils (EOs), there is growing interest in their use as novel alternative synthetic insecticides [4].

The genus *Cymbopogon* (family Poaceae) comprises about 144 species, widely spread throughout the tropical and sub-tropical regions [5]. Plants in the genus *Cymbopogon* are good sources of EOs and are rich in bioactive compounds, including alcohols, aldehydes, and phenolic compounds. The chemical compositions of *Cymbopogon* spp. EOs, such as those from *Cymbopogon citratus* [6,7,8], *Cymbopogon distans* [9], *Cymbopogon flexuosus* [10], *Cymbopogon martinii* [11,12], *Cymbopogon nardus* [13,14,15], *Cymbopogon nervatus* [5,16], *Cymbopogon schoenanthus* [15,17,18], and *Cymbopogon winterianus* [19,20] were assessed in previous studies.

*Cymbopogon* spp. EOs present significant inter/intra-species differences in their chemical compositions. An EO of *C. citratus* from China is reported to be rich in citronellal (38.16%) [7] and geraniol (25.19%) [8], whereas cis-citral (Italy, 59.19%) [21], geranial (Brazil, 50.18%) [6] and neral (Benin, 24.6%) [22] were the major components in *C. citratus* oil in other countries. Similarly, citronellal (22.15–41.7%) [14,15,22,23] and citral (38.75%) [13] are reported as major components in *C. nardus* oil. Piperitone (59.2–71.5%) [15,17], α-eudesmol (17.89%) [18], and cis-p-Menth-2-en-1-ol (28.5%) [24] were the major components in *C. schoenanthus* oil. The major components of *C. flexuosus* oil were neral (30.4%) [10] and geranial (38.44%) [25]. A previous study reported that citronellal (24.0–55.4%) [19,26] was the main constituent in *C. winterianus* oil. Trans-p-mentha-2, 8-dien-1-ol (20.70%) [11] and geraniol (76.9%) [12] are reported as major constituents in *C. martinii* oil. Trans-p-mentha-1 (7), 8-dien-2-ol (32.6%) [16] and trans-p-mentha-2, 8-dien-1-ol (13.6%) [5] are reported as major components in *C. nervatus* oil.

According to previous reports, *Cymbopogon* EOs present good insecticidal activity against vector pests, such as *Anopheles funestus* [27], *Aedes aegypti* [28,29], *Aedes albopictus* [30], *Cochliomyia hominivorax* [31], *Culex quinquefasciatus* [32], *Haemaphysalis longicornis* [33], *Musca domestica* [34], *Rhipicephalus microplus* [35], and *Sarcoptes scabiei* [36]; storage pests, including *Acanthoscelides obtectus* [37], *Callosobruchus maculatus* [25,38,39], *Dinoderus porcellus* [22], *Rhyzopertha dominica* [40], *Sitophilus granaries* [41], *Sitophilus oryzae* [42,43,44], *Sitophilus zeamais* [45,46], *Tenebrio molitor* [47], *Trogoderma granarium* [48], *Tribolium castaneum* [49,50,51,52,53], and *Ulomoides dermestoides* [23,54]; and agricultural insect pests, such as *Bemisia tabaci* [55], *Euprosterna elaeasa* [56], *Megalurothrips sjostedti* [57], *Phthorimaea operculella* [58], *Spodoptera exigua* [59], *Spodoptera frugiperda* [60,61], *Trichoplusia ni* [62,63], and *Tuta absoluta* [64].

However, there exist almost no reports on the vapor toxicity efficacy of *Cymbopogon* spp. EOs against *Reticulitermes flaviceps*. Thus, the objectives of the present study were to: (1) evaluate the constituents of lemongrass EO; (2) evaluate the vapor activity of lemongrass EO and its major constituent against *R. flaviceps* in terms of their behavioral effect on the walking and gripping capability of workers; and (3) investigate the effects of citral on the chemical structure of chitin in *R. flaviceps*.

## 2. Materials and Methods

### 2.1. Insects

Two colonies of subterranean termite *R. flaviceps* were collected at Linglong Mountain in Lin’an, Hangzhou, and three colonies of *R. flaviceps* were collected from ZAFU campus, and reared with water and Mason’s pine (*Pinus massoniana* L.) in the laboratory. We selected healthy and active workers of uniform size for further experiments.

### 2.2. Lemongrass EO and the Constituents

Lemongrass EO was obtained from the Moellhausen Flagship Store (Shanghai, China) and its major constituent, citral (96%), was purchased from TCI Shanghai (Shanghai, China) and kept at 4 °C until further use.

### 2.3. GC-MS Analysis

The lemongrass EO was determined using a gas chromatograph (Agilent 6890A, Santa Clara, CA, USA), equipped with a mass spectrometer detector (Agilent 5975C, Santa Clara, CA, USA). The injector temperature was set at 250 °C, the oven temperature was programmed at 50–250 °C (10 °C/min), the He carrier gas flow was 1.0 mL/min (split ratio of 1:50), and a sample volume of 1.0 μL was injected. Compounds were identified using NIST11.LIB, through comparison of retention indices (RI) with respect to those reported in the Adams [65] library.

### 2.4. Vapor Toxicity

To conduct fumigations [3], filter paper strips were stuck to the lids of 1 L glass jars and 0.12–0.22 μL of lemongrass EO; its major component citral, or (acetone as a control) was added. Twenty healthy workers were put into a glass bottle, the bottle cap was quickly closed, and a moist filter paper was placed on the bottom of the bottle as food. The experiment was repeated three times with three colonies, and the glass jars were kept at 25 ± 1 °C and 80% RH. A portion of moistened filter paper was placed at the bottom of the bottle for water and food. After 24 h, the number of dead termites was observed and recorded.

### 2.5. Behavior Effect

#### 2.5.1. Walking Behavior

*R. flaviceps* workers were anesthetized with carbon dioxide for 5 s, then 1 μL of the lemongrass EO or citral was applied to their antennae. The treated workers were transferred to petri dishes lined with moist filter paper and, after 2 h, we observed their walking ability. Workers who could walk continuously for more than 5 s were considered to have normal walking ability, and the number of workers with normal walking ability was recorded. Acetone treatment was used as control, and three replicates with three colonies of 20 workers were used for each dose.

#### 2.5.2. Gripping Behavior

*R. flaviceps* workers were anesthetized with carbon dioxide for 5 s, then 1 μL of the lemongrass EO or citral was applied to their antennae. The treated workers were transferred to petri dishes lined with moist filter paper. After 2 h, the petri dish was covered with a piece of filter paper and gently inverted (with the mouth facing downward) for 5 s. Then, the petri dish was gently inverted back up again, and we immediately recorded the number of workers stuck to the paper. Acetone treatment was used as control, and three replicates with three colonies of 20 workers were used for each dose.

### 2.6. Effect of Chitin Structural Chemistry

#### 2.6.1. Insect Treatment

Fifty healthy and active adult workers were placed in glass vials and treated with a sub-lethal concentration (LC_20_ = 0.16 μL/L) of citral for 6 h, as described in Section 2.4.

#### 2.6.2. Chitin Extraction

Chitin was extracted according to the procedure of Shah et al. [66], including three steps of demineralization, deproteinization, and decolorization. In brief, samples were first demineralized by treatment with 1 M HCl for 20 min at 100 °C. Then, the washed samples were deproteinized with 1 M NaOH solution for 24 h at 80 °C. Finally, the obtained chitin samples were decolorized through incubation in a mixture of chloroform, methanol, and water (1:2:4, *v*/*v*/*v*) for 20 min. The percentage of chitin weight was calculated based on the formula:

$$W=\frac{W2}{W1}\times 100,$$

where *W*_1_ represents the weight of the raw sample and *W*_2_ represents the weight of the chitin.

#### 2.6.3. Fourier Transform Infrared Spectroscopy (FTIR)

Fourier transform infrared spectroscopy (Prestige-21, SHIMADZU, Kyoto, Japan) was conducted to measure absorbance values between 250 and 4000 cm^−1^. Before measurement, a 1 mg sample of chitin was added to 100 mg of purified potassium bromide (KBr) powder. The effects of citral treatments on the molecular structure and composition of chitin were evaluated by observing the changes in the infrared band. Each experiment was repeated three times.

#### 2.6.4. Thermogravimetric Analysis (TGA)

Citral-treated chitin samples were analyzed using a NETZSCH TG 209 F1 Libra thermal gravimetric analyzer (Selb, Germany). A 10 mg chitin sample was heated from 30 °C to 500 °C in nitrogen at a rate of 10 °C/min. The effect of citral on the thermal stability of the extracted chitin was analyzed by thermogravimetric (TG) and differential thermogravimetric (DTG) analyses.

#### 2.6.5. X-ray Diffraction (XRD)

X-ray diffraction spectra were obtained using an X’Pert-Pro MPD X-ray diffractometer (Almelo, Holland). Data were collected on a copper target at a scan rate of 1°/min with a scan angle of 5–40°. The crystallinity index was calculated using the following equation:

$$C_{r}I=\frac{I110-Iam}{I110}\times 100,$$

where *I*_110_ is the maximum intensity at 2θ ≅ 20° and *I_am_* is the intensity of amorphous diffraction at 2θ ≅ 16°.

#### 2.6.6. Differential Scanning Calorimetry (DSC)

DSC analysis of 10 mg control and citral-treated chitin samples was conducted using a NETZSCH DSC 204F1 apparatus (Selb, Germany).

### 2.7. Statistical Analysis

Toxicity data were subjected to probit analysis in order to estimate the LC_50_ values of lemongrass EO and the major constituent. The mortality rate, chitin content, and walking and gripping ability data were subjected to one-way ANOVA and Tukey’s HSD test (*p* < 0.05).

## 3. Results

### 3.1. Chemical Composition of Lemongrass EO

The chemical compositions of the lemongrass *s* EO are shown in Table 1. Nine components comprising 96.84% of the total lemongrass EO composition were identified. cis-citral (36.51%), trans-citral (31.42%), and geraniol (8.78%) were identified as major components of the lemongrass oil.

### 3.2. Vapor Activity of Lemongrass EO and the Major Constituent

The efficacy of lemongrass EO against *R. flaviceps* was significantly increased with increasing concentration (F = 32.73; df = 5, 12; *p* < 0.001; Table 2). The LC_50_ value of lemongrass EO against *R. flaviceps* showed high toxicity, with a value of 0.328 (Table 2). Additionally, citral also showed a significant variation in vapor toxicity at different concentrations (F = 184.85; df = 5, 12; *p* < 0.001; Table 2), with an LC_50_ of 0.177 (Table 2).

### 3.3. Effects of Lemongrass EO and the Major Constituent on Walking Behavior

**Figure 1:**
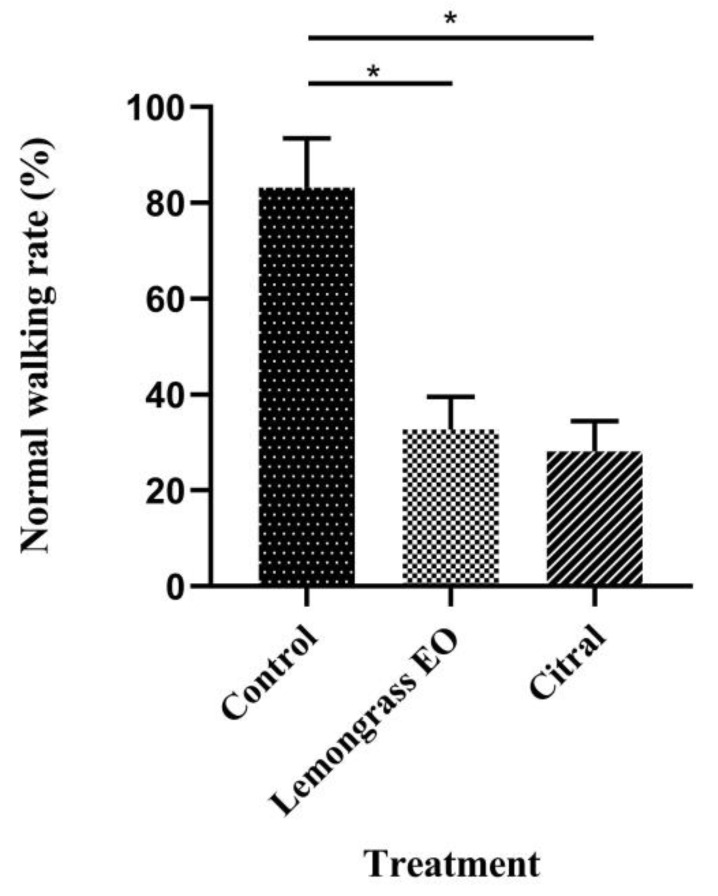
Effect of lemongrass EO and its major constituent on the walking behavior of *R. flaviceps* workers. Means (±SD) values with * show significant differences (*p* < 0.01), according to Tukey’s HSD test.

Lemongrass EO and its major constituent significantly affected the walking ability of *R. flaviceps* workers, compared with controls (*p* < 0.01; Figure 1). In the control group, the workers walked quickly and lasted for a long time, while the walking rate of the workers in all treatment groups was significantly slowed down to where the workers could not walk continuously or even completely. In general, the walking ability of the citral-treated termites was lower than that of those treated with lemongrass EO.

### 3.4. Effects of Lemongrass EO and Its Major Constituent on Gripping Behavior

**Figure 2:**
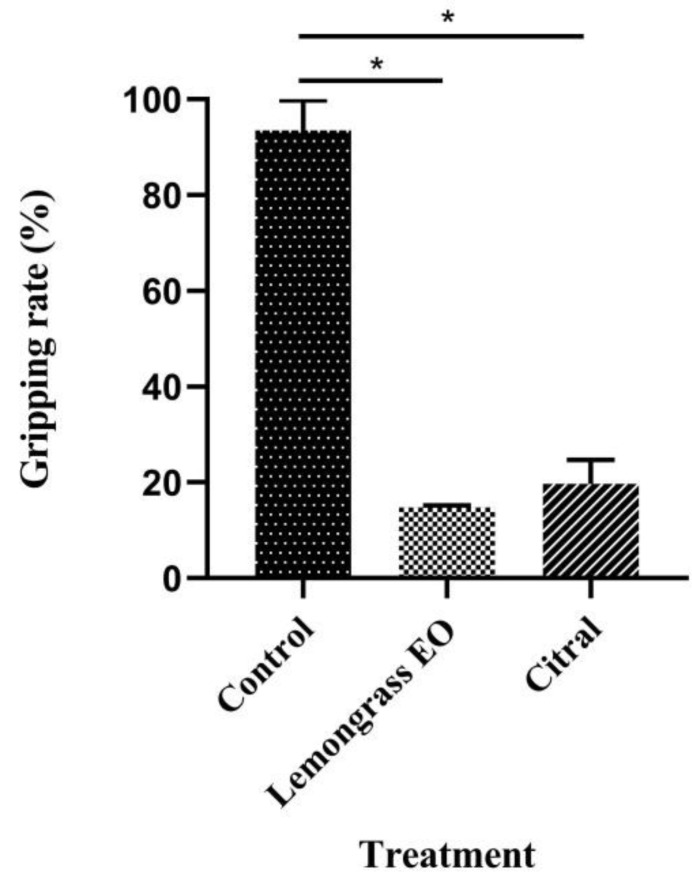
Effect of lemongrass EO and its major constituent on the gripping behavior of *R. flaviceps* workers. Mean (±SD) values with * show significant differences (*p* < 0.01), according to Tukey’s HSD test.

The EO of lemongrass and its major constituent significantly affected the gripping ability of *R. flaviceps* workers, compared with controls (*p* < 0.01; Figure 2), where their gripping ability ranged from 11.84 to 22.02%.

### 3.5. Chitin Content

**Figure 3:**
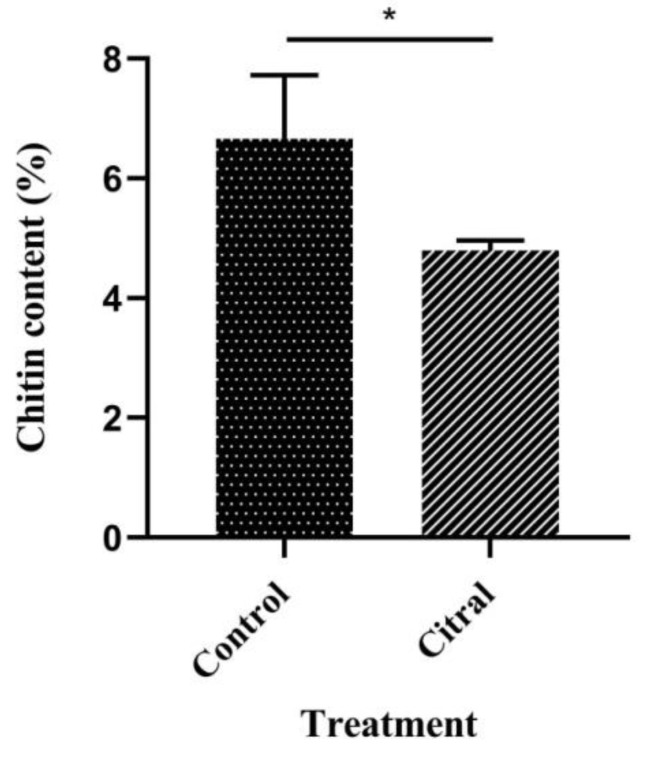
Citral decreases chitin content of *R. flaviceps* workers. Mean (±SD) values with * show significant difference (*p* < 0.01), according to Tukey’s HSD test.

In the present study, the chitin content in the control and citral-treated groups of *R. flaviceps* workers was investigated. As shown in Figure 3, citral (2.91%) led to significantly lower chitin content in *R. flaviceps* workers, compared with the control (6.67%; *p* < 0.01), indicating that citral decreased the chitin content.

### 3.6. Fourier Transform Infrared Radiation

**Figure 4:**
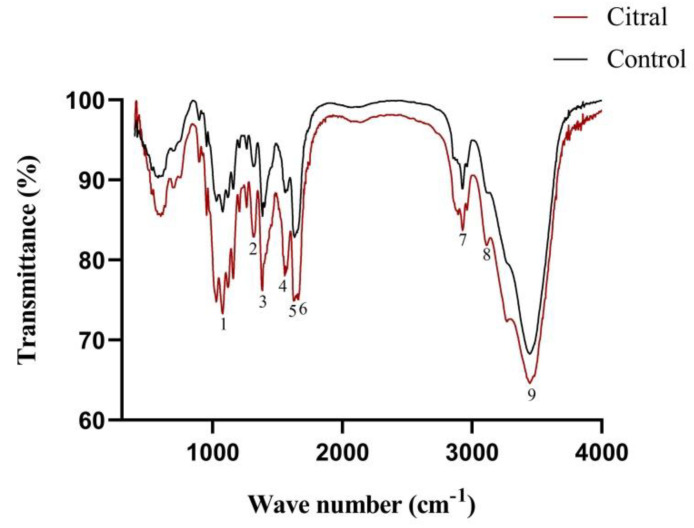
FTIR spectra of α-chitin from *R. flaviceps* in the control and citral-treated groups.

The FTIR spectra are presented in Figure 4 and Table 3. Significant changes in chitin chemical structure properties were observed in the citral-treated group. The chitin chemical structures of the control and citral-treated *R. flaviceps* workers presented three important amide bands at 1560, 1630, and 1656 cm^−1^, corresponding to N–H bending and C–N stretching (amide II), C=O secondary amide stretching (amide I) and C=O secondary amide stretching (amide I), respectively.

### 3.7. Thermogravimetric Analysis

**Figure 5:**
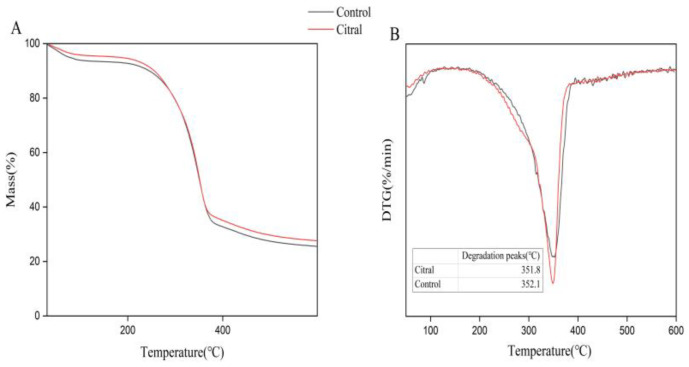
(**A**) TG curves of chitin in the control and citral-treated groups; (**B**) DTG profiles of the chitin of *R. flaviceps* in the control and citral-treated groups.

The thermal stability and degradability of chitin were analyzed by thermogravimetric analysis (TGA; Figure 5). As shown in Figure 5A, three degradation processes occurred: the first one occurred at 30–110 °C, with a weight loss of 5.5%. The second degradation process occurred at 280–370 °C; the degradation rate in this process was accelerated, and the weight loss was 67.8%. Finally, the third degradation process occurred at 403–500 °C, and the degradation tended to be stable in this process. Figure 5B shows the maximum degradation rates of chitin content by citral. The DTG_max_ of control chitin was at 352.1 °C, while the DTG_max_ of citral-treated chitin was at 351.8 °C, indicating that the thermal stability of chitin was affected by citral treatment.

### 3.8. X-ray Diffraction

**Figure 6:**
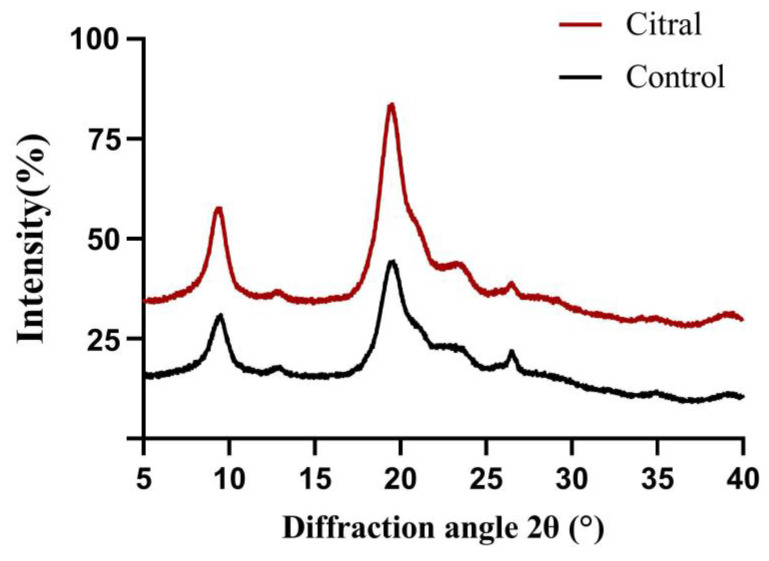
Comparison of X-ray powder diffractograms of the chitin of *R. flaviceps* in the control and citral-treated groups.

The α-crystalline structure of chitin samples was analyzed by X-ray diffraction. As shown in Figure 6, the crystal reflection peaks were located at 9 and 19°. The chitin crystallinity indices of the control and citral-treated samples were 60.8 and 41.2%, respectively. The results indicate that citral destroyed the chemical structure of chitin to varying degrees.

### 3.9. Differential Scanning Calorimetry

**Figure 7:**
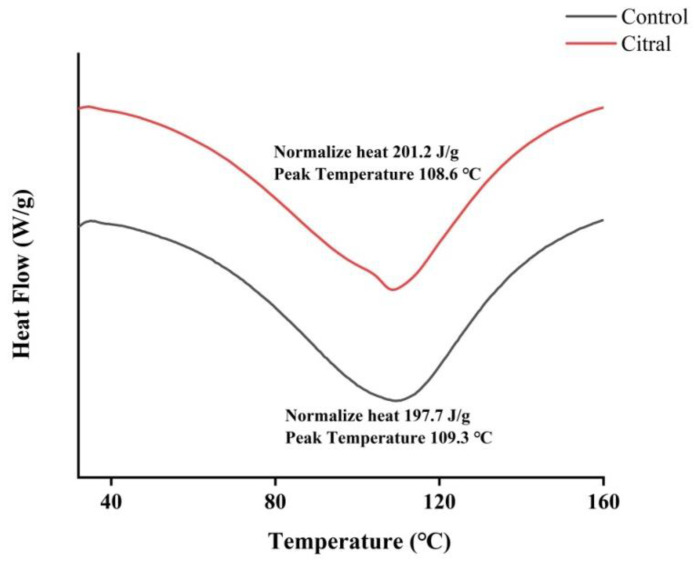
DSC thermogram of the chitin of *R. flaviceps* in the control and citral-treated groups.

The thermal stability of the chitin samples was analyzed by differential scanning calorimetry. As shown in Figure 7, the heat value of the control group was 201.2 J/g, which was significantly higher than that of the treatment group. The maximum decomposition temperature was 109.3 °C in the control group and 108.6 °C in the citral-treated group. These results indicate that treatment with citral changed the thermal stability of chitin, which was consistent with the results of the previous two tests.

## 4. Discussion

The findings presented here reveal that the main components of lemongrass EO are cis-citral (36.51%), trans-citral (31.42%), and geraniol (8.78%); see Table 1. In general, citral (i.e., a mixture of cis- and trans-citral) was the major component of *C. citratus* EO, in agreement with the results of Boukhatem et al. [67], Pinto et al. [68], Feriotto et al. [21], Brugger et al. [69], Manh et al. [28], Soonwera and Sittichok [30], Aungtikun et al. [34], and Loko et al. [22], with slight differences in the relative content. However, previous studies also show that citronellal [7] and geraniol [8] are the major components of *C. citratus* EO, which is not in accordance with our results (Table S1). Genotypic variations, cultivation techniques, extraction method, and agricultural and environmental conditions can influence the chemical composition of plant EOs of the same species [30,34].

In this study, the results clearly demonstrate that the lemongrass EO had insecticidal efficacy against *R. flaviceps* (Table 2). This is in agreement with Xie et al. [70], who demonstrated the antitermitic activity of *Syzgium aromaticum* EO against *R. chinensis* (LC_50_ = 12.5 μg/g) after 7 d. Studies also reported the effectiveness of *Eugenia caryophyllata* EO against *Coptotermes formosanus* [71], *R. speratus* [72], and *Odontotermes obesus* [73]. Similarly, Pandey et al. [74] also reported the antitermitic activity of *Sy. aromaticum* EO on *O. assamensis*. Yang et al. [3] recently demonstrated that the LC_50_ value of spearmint EO against *R. dabieshanensis* was 0.194 μL/L.

There are no previous studies on the insecticidal activities of lemongrass EO against *R. flaviceps*; however, there are previous reports on the insecticidal potential of *C. citratus* EO. In the previous reports, *C. citratus* EO exhibited insecticidal activities against a variety of pest insects belonging to the orders Hemiptera [55], Coleoptera [75], Lepidoptera [59,60], and Diptera [28,30]. These bioassay results demonstrate that *C. citratus* EO has a significant insecticidal effect, which is worthy of further development in the future.

Our results demonstrate that the lemongrass EO and its major component, citral, had strong vapor activity in *R. flaviceps*. Similar results were obtained for citral (LC_50_ of 0.01 μL/L), in terms of its good termiticidal activity against *R. chinensis* [76]. Additionally, citral is shown to possess strong vapor toxicity against *M. domestica* (LC_50_ = 0.74 μL/L) [77]. Similarly, Lee et al. [78], Palacios et al. [79], and Kumar et al. [80] found that citral presents an insecticidal effect against *M. domestica*. The results of the above studies suggest that citral presents similar trends in toxicity for various insect species.

Interestingly, not only did lemongrass EO and its major component show excellent fumigation activity against *R. flaviceps* workers, but they also significantly affected the walking and gripping ability of workers when applied to their antennae. Similarly, Zhang et al. [81] reported that *Citrus paradisi* EO and its main compounds significantly suppressed the walking and gripping abilities of *Solenopsis invicta* workers. Fu et al. [82] also reported that camphor EO affects attacking, feeding, and climbing behaviors in *S. invicta* workers. Any reduced walking, gripping, and climbing abilities of social insects results in their inability to adapt to the environment. The above conclusions indicate that the vapor activity of *C. citratus* EO and citral may be related to their effects on the social behaviors of the insects.

We observed a significant decrease in chitin content in the citral-treated group. Further, through the FTIR analysis results, it was found that the chitin of *R. flaviceps* exists in the α-form (α-chitin). This is consistent with the previous reports of Zhang et al. [83] and Shah et al. [66], who showed that most insect chitin exists in the α-form. Crystalline α-chitin usually exhibits FTIR bands in the range of 1550, 1620, and 1650 cm^−1^ [66,84]. Based on this, it was found that citral treatment induced changes in the chemical structure of *R. flaviceps* chitin.

In the TGA analysis plot of the extracted chitin structures, mass loss was seen in two steps (Figure 5). In the first step, 8% to 10% weight loss was recorded due to water vaporization [66,85,86]; meanwhile, in the second step, the decomposition could be attributed to the decomposition of chitin saccharide structures [87]. In a previous study, the DTG_max_ decomposition temperature of α-chitin extracted from *Sitotroga cerealella* was 388 °C [66]. In addition, Wang et al. [88] found that the DTG_max_ decomposition temperature of α-chitin extracted from organisms such as crab, shrimp, krill, and crayfish ranged between 350 and 381 °C. Therefore, the DTG_max_ disintegration temperatures of α-chitin structures extracted from different organisms are different. According to Aranaz et al. [89], the decomposition temperature of chitin affects its usefulness. In the present study, the disintegration temperature of chitin extracted from the control and citral-treated groups was similar to that reported in other studies, varying between 385 and 389 °C [66]. TGA showed that treatment with citral slightly reduced the thermal stability of chitin, which may also cause changes in its chemical structure and reduce its crystallinity. These differences may be due to the N-acetylated polymer units of chitin being more stable in the control group than in the treated group. This is consistent with the results of the previous FTIR analysis. Zia et al. [90] reported that chitin has a highly ordered crystal structure. The XRD analysis results in this study were consistent with previous findings [66,91]. As shown in Figure 6, a broad signal centered at 2θ = 9° was presented, which was attributed to the GlcNAc sequence [92]. Furthermore, due to the GlcN sequence, the intensity of the broad signal was centered at 2θ = 19° [66].

In addition, it is known in the literature that most of the EOs and their major components can exert their toxic efficacy on insects notably through inhibition of P450 cytochromes (CYPs), GABA receptors, octopamine synapses, and the inhibition of acetylcholinesterase (AchE) [3]. Alves et al. [38] found that lemongrass EO and citral showed AChE inhibitory efficacy in *Callosobruchus maculatu*. Decreased AChE activity causes a direct change in insect behavior, such as flight, copulation, and oviposition, as well as changes in many other biological processes of insects [38]. Therefore, the exact toxicity mode of action and target of lemongrass EO and citral against the tested *R. flaviceps* in this study need to be revealed and confirmed by further experiments.

## 5. Conclusions

In this study, we clearly demonstrated that lemongrass EO and citral show potential vapor toxicity against *R. flaviceps*. The higher vapor toxicity of the lemongrass EO can be ascribed to its major constituent, citral. In addition, the lemongrass EO and citral significantly inhibited the walking and gripping abilities of *R. flaviceps* workers. Analysis results indicate that citral significantly reduced the content of chitin and changed the chemical structure in *R. flaviceps*. As such, we expected that citral has great potential for development into a new type of termite control agent. Future research should include formulation development, in order to determine whether sustained-release formulations can be designed, which may be delivered by termite workers to the nest to achieve the vapor of other individuals in the nest, as well as study the effects of temperature on such a slow-release application.

## Figures and Tables

**Table 1:** Chemical composition of lemongrass EO.

No.	Constituents	RI ^a^	RI ^b^	%
1	α-Pinene			1.83
2	β-Pinene	935	932	0.64
3	Limonene	979	977	2.46
4	1,8-Cineole	1029	1025	6.52
5	Linalool	1038	1036	-
6	trans-Citral	1097	1095	31.42
7	Geraniol	1240	1235	8.78
8	Citronellyl formate	1250	1249	-
9	cis-Citral	1277	1271	36.51
10	Geranyl acetate	1316	1312	4.85
11	Neryl acetate	1352	1350	-
12	Caryophyllene	1365	1359	3.83
		1419	1417	
	Total identified (%)			96.84

^a^ RI, linear retention indices on HP-5MS column, experimentally determined using homologue series of n-alkanes. ^b^ Retention indices (RI) values taken from Adams [65].

**Table 2:** Mortality rate at 24 h, lethal concentration for 50% and 90% mortality of lemongrass EO and citral against *R. flaviceps*.

Treatment	Conc. (μL/L)	Mortality (%) ± SD at 24 h	LC_50_ ^a^ (LCL-UCL)	LC_90_ ^a^ (LCL-UCL)	Regression	χ^2 b^ (d.f. = 4)	R^2 c^
Lemongrass EO	0.14 0.16	0 e * 3.3 ± 2.9 e	0.328 (0.222–0.391)	0.595 (0.524–0.720)	y = 4.8167x − 0.655	3.685 n.s	0.982
	0.18	30.0 ± 5.0 d					
	0.20	31.7 ± 5.0 d					
	0.22	38.3 ± 7.6 cd					
Citral	0.14 0.16	6.7 ± 2.9 e 15.0 ± 5.0 e	0.177 (0.171–0.185)	0.214 (0.203–0.233)	y = 12.417x − 1.7383	32.464 n.s	0.973
	0.18	48.3 ± 2.9 c					
	0.20	80.0 ± 10.0 b					
	0.22	98.3 ± 2.9 a					

^a^ LC_50_, LC_90_ = lethal concentration for 50% and 90% mortality with 95% confidence limit; LCL = lower confidence limit; UCL = upper confidence limit. ^b^ χ^2^ = chi-square value with α = 0.05. ^c^ R^2^ = regression coefficient. * The means in each row against *R. flaviceps* that are followed by different letters are significantly different (*p* < 0.05, by ANOVA and Tukey’s HSD Test). d.f. = degrees of freedom. n.s. = not significant (*p* > 0.05).

**Table 3:** Characteristics and variations in bands in the FTIR spectra of the chitin of *R. flaviceps* treated with citral and control insects.

No.	Wave Number (cm^−1^)	Functional Group and Vibration Modes	Band Assignment	Control	Citral
1	1050	C–O asym. stretch in phase ring	-	88.21	77.54
2	1315	CH_2_ wagging	Amide III, components of proteins	91.65	82.77
3	1385	C–H bend, CH_3_ sym. Deformation	-	85.77	76.88
4	1560	N–H bend, C–N stretch	Amide II	88.81	79.32
5	1630	C=O secondary amide stretch	Amide I	82.92	75.05
6	1656	C=O secondary amide stretch	Amide I	93.78	75.28
7	2932	CH_3_ sym. stretch and CH_2_ asym. stretch	Aliphatic compounds	89.53	84.08
8	3111	N–H secondary amine asym. stretch	Amide II	88.46	81.86
9	3440	O–H hydroxyl stretching	-	68.31	64.70

## Data Availability

The dataset utilized in this study is available upon request.

## Supplementary Materials

The following supporting information can be downloaded at: www.mdpi.com/article/10.3390/insects13090812/s1, Table S1: Major components of *Cymbopogon citratus* EO from different origins previously reported.
