# Supplementary material for: Toxicity, Behavioral Effects, and Chitin Structural Chemistry of Reticulitermes flaviceps Exposed to Cymbopogon citratus EO and Its Major Constituent Citral
Source: Insects. 2022 Sep 6;13(9):812. doi: 10.3390/insects13090812 (PMC9501940; doi:10.3390/insects13090812)
Supplement: Supplementary file 1 [file insects-13-00812-s001.zip › insects-1864194-supplementary.pdf]

Table S1. Major components of *Cymbopogon citratus* EO from different origins previously reported.

Cymbopogon citratus	*Origin*	Major components	Ref.
C. citratus	*China*	cis-Citral (36.51%), trans-citral (31.42%), geraniol (8.78%), 1,8-cineole (6.52%)	Res
C. citratus	*Brazil*	Geranial (50.18%), neral (31.53%), myrcene (1.59%), geraniol (1.40%)	[6]
C. citratus	*China*	Citronellal (38.16%), geraniol (19.39%), citronellol (17.18%), d-limonene (5.16%).	[7]
C. citratus	*China*	Geraniol (25.19%), citronellal (21.64%), citronellol (14.71%), cyclohexanemethanol, 4-ethenyl-a,a,4-trimethyl-3-(1-methylethenyl)-, (1 R,3 S,4 S)- (14.09%)	[8]
C. citratus	*Ecuador*	cis-citral (59.17%), β-pinene (22.47%), cis-verbenol (6.14%), nerol (4.98%)	[21]
C. citratus	*Benin*	Neral (26.64%), geranial (23.46%), β-pinene (21.90%), nerol (8.54%)	[22]
C. citratus	*Vietnam*	Geranial (49.3%), neral (36.2%), β-myrcene (7.8%), geraniol (4.1%)	[28]
C. citratus	*Thailand*	Geranial (45.37%), neral (24.78%), 1,8-cineole (10.56%), geraniol (4.71%)	[30]
C. citratus	*Thailand*	Geranial (45.4%), neral (24.8%), 1,8-Cineole (10.6%), geraniol (4.7%)	[34]
C. citratus	*Brazil*	Geranial (46.83%), neral (34.10%), myrcene (12.57%), (E)-isocitral (1.76%)	[38]
C. citratus	*Algeria*	Geranial (42.16%), neral (31.52%), β-myrcene (7.45%), geranyl acetate (4.3%)	[67]
C. citratus	*Cuba*	Geranial (51.14%), neral (35.21%), myrcene (6.52%), geraniol (2.23%)	[68]
C. citratus	*Brazil*	Neral (31.5%), citral (26.1%), Nonan-4-ol (6.54%), camphene (5.19%)	[69]
